# Rational design of mechanically robust Ni-rich cathode materials via concentration gradient strategy

**DOI:** 10.1038/s41467-021-26290-z

**Published:** 2021-10-15

**Authors:** Tongchao Liu, Lei Yu, Jun Lu, Tao Zhou, Xiaojing Huang, Zhonghou Cai, Alvin Dai, Jihyeon Gim, Yang Ren, Xianghui Xiao, Martin V. Holt, Yong S. Chu, Ilke Arslan, Jianguo Wen, Khalil Amine

**Affiliations:** 1 Chemical Sciences and Engineering Division, Argonne National Laboratory, Lemont, IL 60439 USA; 2 Center for Nanoscale Materials, Argonne National Laboratory, Lemont, IL 60439 USA; 3 National Synchrotron Light Source II, Brookhaven National Laboratory, Upton, NY 11973 USA; 4 X-ray Science Division, Advanced Photon Sources, Argonne National Laboratory, Lemont, IL 60439 USA; 5 Material Science and Engineering, Stanford University, Stanford, CA 94305 USA

**Keywords:** Energy, Batteries, Batteries, Electrochemistry

## Abstract

Mechanical integrity issues such as particle cracking are considered one of the leading causes of structural deterioration and limited long-term cycle stability for Ni-rich cathode materials of Li-ion batteries. Indeed, the detrimental effects generated from the crack formation are not yet entirely addressed. Here, applying physicochemical and electrochemical ex situ and in situ characterizations, the effect of Co and Mn on the mechanical properties of the Ni-rich material are thoroughly investigated. As a result, we successfully mitigate the particle cracking issue in Ni-rich cathodes via rational concentration gradient design without sacrificing the electrode capacity. Our result reveals that the Co-enriched surface design in Ni-rich particles benefits from its low stiffness, which can effectively suppress the formation of particle cracking. Meanwhile, the Mn-enriched core limits internal expansion and improve structural integrity. The concentration gradient design also promotes morphological stability and cycling performances in Li metal coin cell configuration.

## Abstract (web summary)

Mechanical integrity issues are one of the main causes of limited long-term cycle stability for Ni-rich cathode materials. Here the authors analyse the roles of cobalt and manganese and utilise a concentration gradient design to mitigate these issues.

## Introduction

Nickel-rich-layered oxides (LiNi_*x*_Mn_*y*_Co_*z*_O_2_ Ni-rich NMC; *x* ≥ 0.7) have been highlighted as the most promising cathode candidate for next-generation lithium-ion batteries by virtue of their accessible capacity and low cost^1–5^. Despite the benefits to capacity however, increasing Ni content significantly exacerbates capacity degradation, which greatly restricts large-scale commercialization of Ni-rich NMC^6–8^. In order to surpass these limitations, much effort has been made to investigate the intrinsic properties of Ni-rich NMC and mechanisms for fast capacity fade^9–13^. Gathered evidence reveals that the capacity degradation of Ni-rich NMC is typically attributed to surface chemical instability and mechanical destruction in the form of microcracks^14–19^. Strategies to resolve surface chemical instability that usually accompany parasitic reactions, irreversible phase transition, and electrolyte decomposition include the use of coatings and other surface protection techniques^13,16,20–23^. Although these surface protections have shown promise for stabilizing the surface chemistry, negligible improvements to the mechanical properties and no reductions in particle cracking have been realized. Once cracking occurs, electronic transport pathways will be blocked and fresh surfaces will be exposed to the electrolyte, which reduces the efficacy of protective surface measures and induces additional surface parasitic reactions^24–26^. As a result, particle crack suppression is of particular significance for cycle stability in Ni-rich NMC and for most layered oxide cathodes^27^.

It has been revealed from recent operando studies that anisotropic volume change in primary particles and lattice strain initiate the mechanical destruction of secondary particles^19,24–26,28^. This heterogeneous volume change breaks contact between randomly oriented primary particles and when combined with lattice strain, leads to chemomechanical breakdown in the form of intergranular and intragranular cracking, respectively^10,29–32^. Cracking becomes more pronounced in Ni-rich cathodes because deeper delithiation produces more severe phase transitions and anisotropic volume change^33^. However, as the demand for high energy density batteries increases, high Ni contents in layered cathodes become unavoidable^34^. Therefore, the most crucial technological challenge for Ni-rich cathodes is the development of methods that can improve their mechanical stability and inhibit particle cracking without sacrifices in capacity. Although Mn and Co are commonplace dopants in Ni-rich cathodes, the intrinsic effect of their compositions on mechanical properties is not well understood^34–36^. The emphasis on Ni content in literature and its associated effects on stability thus far may have resulted in less optimal compositional design solutions^33,34^. Therefore, this knowledge gap necessitates in-depth fundamental investigation to fully understand the mechanical property of Ni-rich cathodes. Understanding the relation of mechanical stability with composition design, especially for Mn and Co, is essential for regulating the mechanical stability of Ni-rich cathodes and resolving capacity degradation caused by particle cracking.

In this work, we systematically investigate the effects of Co and Mn on the mechanical properties of Ni-rich cathodes, regulate the mechanical stability of particles with rational concentration gradient design, and successfully resolve particle cracking in Ni-rich cathodes. Previous concentration gradient NMC cathodes mainly considered Ni content changes from bulk to surface, while here Mn and Co compositions are controlled to investigate their respective mechanical properties and achieve mechanical stability without sacrificing the electrode capacity^37^. Based on comparative studies of two converse composition designs, we demonstrate that gradient cathodes with Co-enriched surfaces exhibit comprehensive advantages over cathodes with Mn-enriched surfaces. With advanced in situ XRD and ex situ morphology measurements, it is understood that Co-enriched surfaces benefit from its lower stiffness, which can effectively expand to relieve particle damage caused by internal expansion and surface compressive stress. Meanwhile, a Mn-enriched core not only limits internal expansion but also increases overall structure reversibility^34^. Together, these two aspects of concentration gradient design significantly enhance the cycle reversibility of Ni-rich NMC. In contrast, concentration gradient design with a Mn-enriched surface and Co-enriched core suffers from severe mechanical strain and greater internal tension imposed on the stiff surface components, which leads to severe particle cracking during repeated volume change. These results re-define the significant effects of Co and Mn on mechanical properties, provide new insights into composition designs that strengthen morphological stability of Ni-rich cathodes, and can serve as essential guidelines to help facilitate structurally and morphologically stable cathode materials in lithium-ion batteries.

## Results

### Investigating the effects of Co and Mn on mechanical properties

**Fig. 1:**
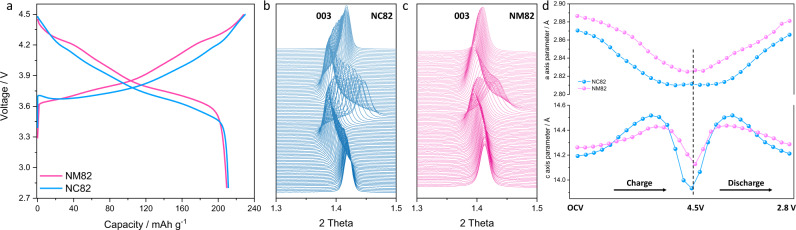
Structure evolution and lattice parameter changes of NC82 and NM82. **a** Charge/discharge profiles of NM82 and NC82 at a current rate of C/10 within a voltage range of 2.8–4.5 V. NM82 and NC82 deliver similar capacities of 209 and 211 mAh g^−1^, respectively. **b**, **c** In situ synchrotron HEXRD results of NC82 and NM82 during the initial cycle in the voltage range of 2.8–4.5 V using a current rate of C/10 (1C = 200 mA g^−1^). **d** The quantitative refinements of lattice parameters of NC82 and NM82.

The stiffness of a material is described by its Young’s modulus (*E*) and is strongly affected by composition and structure. In typical layered oxide cathodes, adjacent oxygen layers electrostatically repel to cause lattice expansion and contraction^30^. This process is generally considered reversible, at least in a single (de)lithiation cycle^28,30,34^, and indicates elastic behavior. The degree of electrostatic repulsion is also highly correlated with residual Li content and its varying screening effects, so we simply define the delithiatiated content as the stress parameter^30^. Lattice parameter evolutions are observed in response to stress can also be directly tracked by in situ XRD measurements. Therefore, by calculating the delithiatiated content and lattice parameter evolution, Young’s modulus for different NMC cathodes can be qualitatively compared, as well as their stiffness. To clarify the individual effects of Co and Mn on mechanical properties, two basic samples, LiNi_0.8_Co_0.2_O_2_ (denoted as NC82) and LiNi_0.8_Mn_0.2_O_2_ (denoted as NM82), are synthesized. As shown in Fig. 1a, these two comparable cathode materials exhibit a similar capacity of 210 mAh g^−1^ at a C/10 charge rate. In situ and ex situ high-energy synchrotron XRD (HEXRD) was conducted to compare the structure evolution during the first cycle. As shown in Fig. 1b–d and Supplementary Figs. 1–4, NC82 undergoes greater lattice expansion and contraction along the *c*-axis as compared to NM82. The lattice parameter changes along the *a-*axis in NM82 and NC82 are similar, suggesting the oxidation state changes of transition metals and the delithiation content in the two samples are very close^30,34^. Therefore, the more pronounced lattice parameter change along the *c-*axis induced by a similar level of stress, indicating that the Co-enriched layered structure with a smaller Young’s modulus is less stiff and, in contrast, the Mn-enriched layered structure (NM82) with a higher Young’s modulus shows greater stiffness during the repeated charge/discharge process. In addition, the Mn-enriched NM82 shows significant peak broadening in the highly delithiated state, which suggests significant increases in microstrain upon delithiation^38^. This is further verified by microstrain calculations using Williamson Hall analysis and Supplementary Fig. 5 shows that the microstrain in NM82 is much larger than that in NC82 at the end of charge. Co and Mn content can also affect cycle performance, which is shown in Supplementary Fig. 6 where NM82 has much better cycle stability than NC82. This is attributed to structure reversibility improvements with Mn substitution and has been confirmed in our previous work^34^. Therefore, these results re-define the understanding between Co/Mn composition designs and mechanical properties to achieve structure stability in Ni-rich cathodes.

### Comparable concentration gradient cathodes and their electrochemical performance

**Fig. 2:**
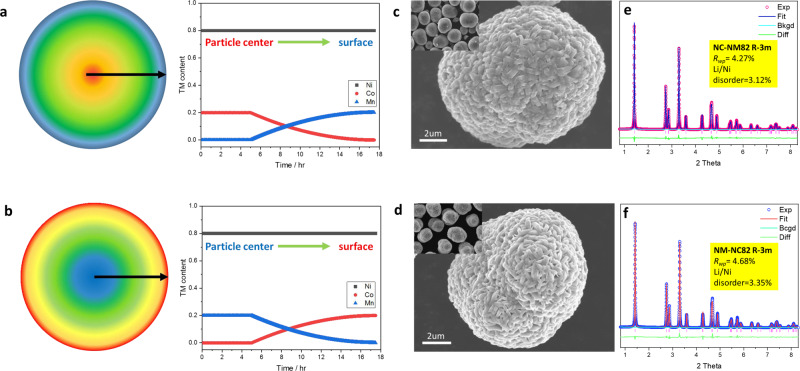
Structural and morphological characterizations of the Ni-rich cathodes. **a** Schematic image of NC-NM82 and the corresponding compositional variation. **b** Schematic image of NM-NC82 and the corresponding compositional variation. **c** SEM image of the NC-NM82 particle. The inserted figure represents a SEM image in a larger area. **d** SEM image of the NM-NC82 particle, the inserted figure represents a SEM image in a larger area. **e** High-energy XRD curve and Rietveld refinement result of NC-NM82. **f** High-energy XRD curve and Rietveld refinement result of NM-NC82.

To clarify the effects of composition variations, two concentration gradient Ni-rich cathode materials with identical average compositions were designed with converse Co/Mn distribution. These designs differ from previous gradient cathode materials, where Ni content always decreases and Mn content increases from the bulk to the surface of the particle^37^, as the two gradient materials maintain a constant high Ni content in the whole particle and utilize varying Co and Mn contents to optimize the structural/mechanical properties and electrochemical performance. As shown in Fig. 2a, b, the composition of 0.5(LiNi_0.8_Co_0.2_O_2_)_bulk_0.5(LiNi_0.8_Mn_0.2_O_2_)_surface_ sample (denoted as NC-NM82) starts with the constant ratio of Ni/Mn/Co (8:0:2, molar ratio) in the particle center, and then gradually changes to Ni/Mn/Co (8:2:0, molar ratio) towards the surface of the particle. The 0.5(LiNi_0.8_Mn_0.2_O_2_)_bulk_0.5(LiNi_0.8_Co_0.2_O_2_)_surface_ sample (denoted as NM-NC82) shows a converse composition design where the central component is Ni/Mn/Co (8:2:0, molar ratio), then gradually changes to Ni/Mn/Co (8:0:2, molar ratio) towards the surface of the particle. Meanwhile, conventional LiNi_0.8_Mn_0.1_Co_0.1_O_2_ (denoted as NMC811) was prepared as a control sample. All three cathode materials were synthesized with a co-precipitation method as described in the “Methods” section.

The overall compositions and morphologies of these three comparable cathode materials were initially analyzed by Inductively coupled plasma-atomic emission spectroscopy (ICP-AES) and scanning electron microscope (SEM). Data in Supplementary Table 1 confirm that the average compositions of the as-prepared samples were identical and close to the expected elemental contents. SEM images (Fig. 2c, d and Supplementary Fig. 7) show that all three cathode materials have uniform secondary-particle morphologies with a similar particle sizes of 12 μm. HEXRD was further conducted to compare the effects of various gradient composition designs on the structural features. As shown in Supplementary Fig. 8, the XRD patterns of as-prepared samples exhibit the characteristic Bragg peaks for a well-crystallized NaFeO_2_ structure with *R-3m* symmetry. Both gradient cathode materials show slightly broadened peaks that are distinct from each other when compared to the conventional NMC811, indicating lattice parameter variations caused by the variable compositions. The Rietveld refinement results (Fig. 2e, f, Supplementary Fig. 9 and Supplementary Table 2) illustrate that the as-prepared samples show a highly uniform atomic occupancy. Only slight Li/Ni disorder is detected in all three samples, indicating their well-crystallized structure.

**Fig. 3:**
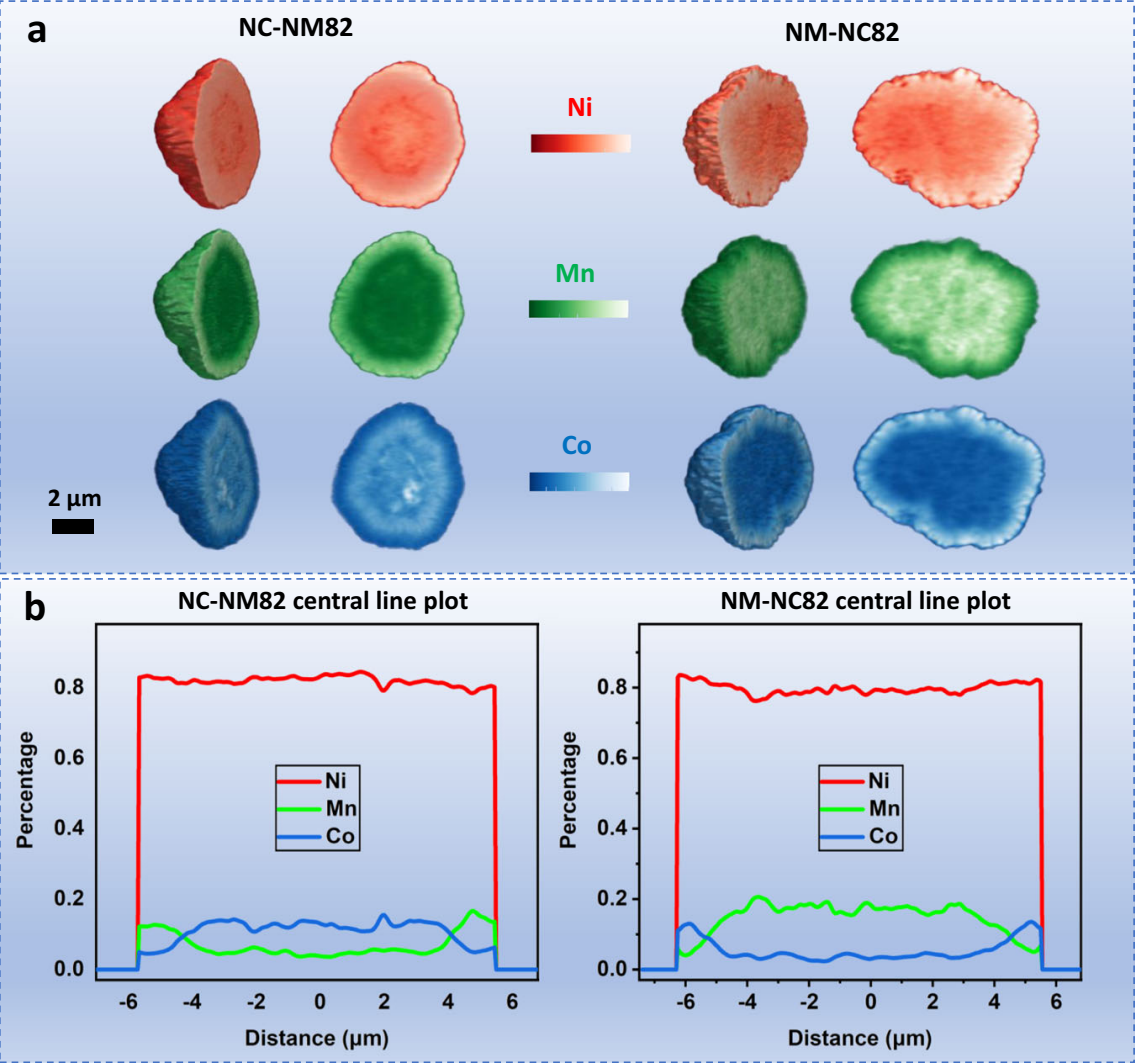
Spatial element distribution of two gradient cathodes. **a** 3D tomography reconstruction from individual particles and spatial element distributions of Ni (red), Mn (green)n and Co (blue), detected by fluorescence-yield scanning probe X-ray microscopy. The element content is reflected by the brightness of the color sets, where the brighter the color, the higher the element content. **b** Central line scanning plots of NC-NM82 and NM-NC82 for the compositional variation.

The actual composition variations within a secondary particle were further investigated by three-dimensional X-ray fluorescence (3D XRF), which is capable of probing the spatial elemental concentration and distribution with high sensitivity. Figure 3a presents the element distribution in virtual cross-sections of the 3D reconstructed particle, and the quantitative analysis was plotted in Fig. 3b and Supplementary Figs. 10 and 11. In general, the average composition of both NM-NC82 and NC-NM82 is close to 80% Ni, 10% Mn, and 10% Co (molar ratio), which is consistent with the ICP-AES results. Importantly, these two cathode materials show gradient changes in Co and Mn distributions but a constant Ni composition. NM-NC82 has an Mn concentrated core and Co gradually increases toward the surface. NC-NM82 shows a converse concentration design, where Co is arranged in the core and Mn gradually increases toward the surface. These results indicate the compositional variation of the two gradient cathode materials is consistent with what was initially designed.

**Fig. 4:**
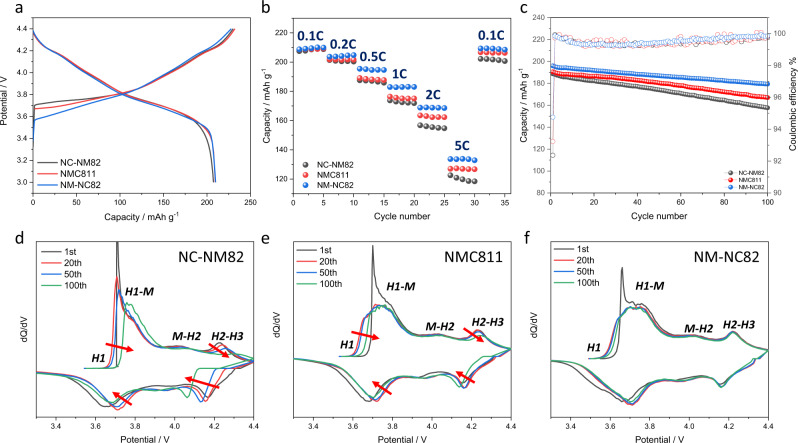
Electrochemical measurements of two gradient cathodes. **a** Initial charge/discharge profile of the NC-NM82, NMC811, and NM-NC82 at 0.1C and a cut-off potential of 4.4 V. **b** Rate capability of the NC-NM82, NMC811, and NM-NC82. **c** Cycling performance of the NC-NM82, NMC811, and NM-NC82 at a current rate of C/2 within a voltage range of 2.8–4.4 V after three activation cycles. **d** d*Q*/d*V* curve at different cycles of NC-NM82. **e** d*Q*/d*V* curve at different cycles of NMC811. **f** d*Q*/d*V* curve at different cycles of NM-NC82.

Given the apparent composition variation of the as-prepared samples, it was of great significance to explore correlations between the electrochemical performance and the gradient composition designs. As illustrated in Fig. 4a, the initial discharge capacities at a current rate of C/10 (1C = 200 mA g^−1^) are recorded as 207, 208, and 208 mAh g^−1^ within a voltage window of 2.8–4.4 V for NC-NM82, NM-NC82, and NMC811, respectively. Moreover, the NM-NC82 exhibits less over-potential, which is attributed to the improved electronic conductivity of the Co-enriched surface. Figure 4b and Supplementary Fig. 12 compared the rate capability of the three cathode materials, where NM-NC82 exhibits the best rate capability owing to their higher electronic conductivity. By contrast, the Mn-enriched surface of NC-NM82 is detrimental to rate performance. This should be attributed to the poor electronic conductivity of the Mn-enriched surfaces. Figure 4c shows the cycling performance of the three cathodes operated at a current of C/2 within a voltage range of 2.8–4.4 V. Capacity retentions over 100 cycles were recorded as 83%, 87%, and 92% for NC-NM82, NMC811, and NM-NC82, respectively. Clearly, the NC-NM82 suffers from faster capacity degradation at this voltage range while the NM-NC82 exhibited the best capacity retention among these three cathode materials. The structural reversibility was further tracked by the d*Q*/d*V* profiles, as shown in Fig. 4d–f. It is evident that all the d*Q*/d*V* peaks of NC-NM82 show synchronous decay and remarkable shifts during cycling, indicating its poor structural reversibility. Here, the severe phase transition process predominantly causes structural deterioration, which is detrimental to reversible Li-ion diffusion and results in capacity loss. NMC811, on the other hand, exhibits improved structure reversibility, judging from the intensity and shift of the d*Q*/d*V* peaks. More notably, the NM-NC82 gradient cathode shows the best structure stability during cycling. The peak shift and intensity decrease of the d*Q*/d*V* peaks are almost negligible. Based on the above electrochemical tests, the NM-NC82 demonstrates comprehensive advantages in cycle performance and rate performance over NC-NM82 and NMC811. More importantly, this further indicates that rational Co and Mn concentration gradient design can effectively enhance the reversibility and cycle performance of Ni-rich cathode materials. This new concentration design essentially differs from previous concentration gradient materials that rely solely on varying Ni composition, representing a major breakthrough in improvements for Ni-rich cathode materials.

### Correlation between composition designs and mechanical/structure stability

**Fig. 5:**
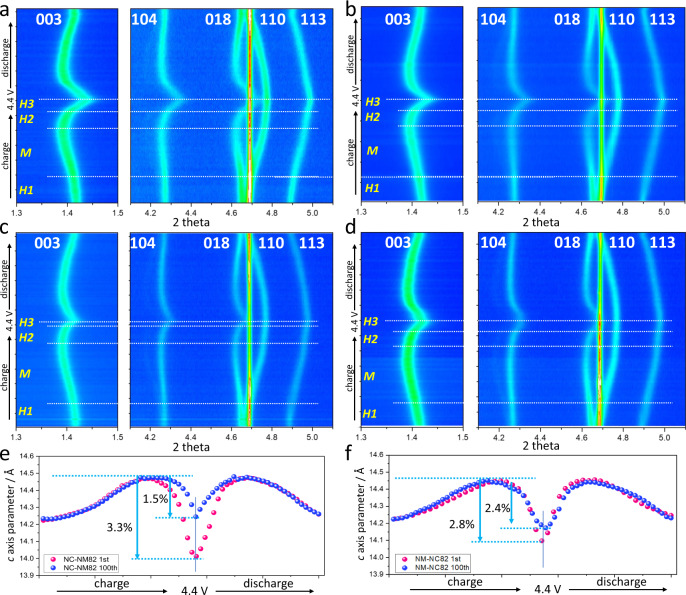
In situ synchrotron HEXRD characterization during the first cycle and the 101th cycle. **a**, **b** Two-dimensional (2D) contour plots of in situ XRD for the structural evolution of NC-NM82 and NM-NC82 during the initial cycle. **c**, **d** 2D contour plots of in situ XRD for the structural evolution of NC-NM82 and NM-NC82 during the 101th cycle. These cells were cycled in the voltage range of 2.8–4.4 V using a current rate of C/10 (1C = 200 mA g^−1^). **e** The *c*-axis lattice parameter changes of NC-NM82 and NC-NM82 during the initial cycle and the 101th cycle obtained from Rietveld refinements. **f** The *c*-axis lattice parameter changes in pristine NM-NC82 and NM-NC82 after 100 cycles obtained from Rietveld refinements.

To understand the correlation between composition designs and structural reversibility, in situ HEXRD measurements are performed on NC-NM82 and NM-NC82 to monitor their real-time structural evolutions. Figure 5 shows the two-dimensional (2D) contour plots for the structure evolutions of NC-NM82 and NM-NC82 cathodes during the first charge–discharge cycle at a current rate of C/10. The structural evolution processes and lattice parameter changes are detailed based on the magnified observation of several characteristic peaks labeled as [003], [018]/[110] pair, and [113]. The result indicates that the two gradient cathodes undergo similar structure evolution processes with slight discrepancy in the degree of lattice parameter changes. As shown in Fig. 5a, b and Supplementary Figs. 13 and 14, the [003] peaks of both NC-NM82 and NM-NC82 shifted to a lower angle up to 4.2 V, beyond which the peak rapidly shifted to a higher angle. The leftward shifts correspond to a series of phase transition processes that increase interlayer spacing, which includes the *H*_*1*_–*M* and *M*–*H*_*2*_ transitions^33,35,39^. Afterwards, the interlayer spacing quickly shrinks during the *H*_2_–*H*_3_ phase transition process^33,35,39^. Such a sharp change in lattice parameter is indicative of lattice strain and subsequently causes particle morphology damage in the form of intragranular microcracks and intergranular microcracks^24,28,31,34^. In view of the comparison between NC-NM82 and NM-NC82, a severe *H*_*2*_–*H*_*3*_ phase transition process occurring in NC-NM82 gradient cathode is detrimental for morphological stability and triggers irreversible phase transition. This process was slightly alleviated by the opposite concentration gradient design of NM-NC82, which is beneficial for structural stabilization during long-term cycling.

In situ HEXRD was further performed to monitor structure evolution in samples after 100 cycles and explicitly verify the distinct structure stability of the two gradient cathode materials. As shown in Fig. 5c and Supplementary Fig. 15, the *H*_2_–*H*_3_ phase transition process of NC-NM82 was significantly weakened after 100 cycles, indicating fast structure degradation. In contrast, NM-NC82 gradient cathode observes negligible degradation of the *H*_2_–*H*_3_ phase transition after 100 cycles (Fig. 5d and Supplementary Fig. 16) and shows improved structural stability. Quantitative analysis of lattice expansion and extraction was obtained from the Rietveld refinement. Figure 5e, f depicts the *c*-axis lattice parameter changes of the first cycle and the 100th cycle for NC-NM82 and NM-NC82, respectively. It is obvious that the *H*_*2*_–*H*_*3*_ phase transition markedly degrades and its reaction potential increases in the NC-NM82 sample, which is consistent with d*Q*/d*V* results. NM-NC82 shows a stable phase transition process, albeit with very slight degradation. It is well accepted that capacity fade in Ni-rich layered cathodes is closely related to the fast degradation of *H*_2_–*H*_3_ phase transition^29,33,35^. Particle cracking and the resulting global occurrence of parasitic reactions are considered as the cause of the rapid capacity loss and accelerated degradation of *H*_2_–*H*_3_ phase transition^33^.

**Fig. 6:**
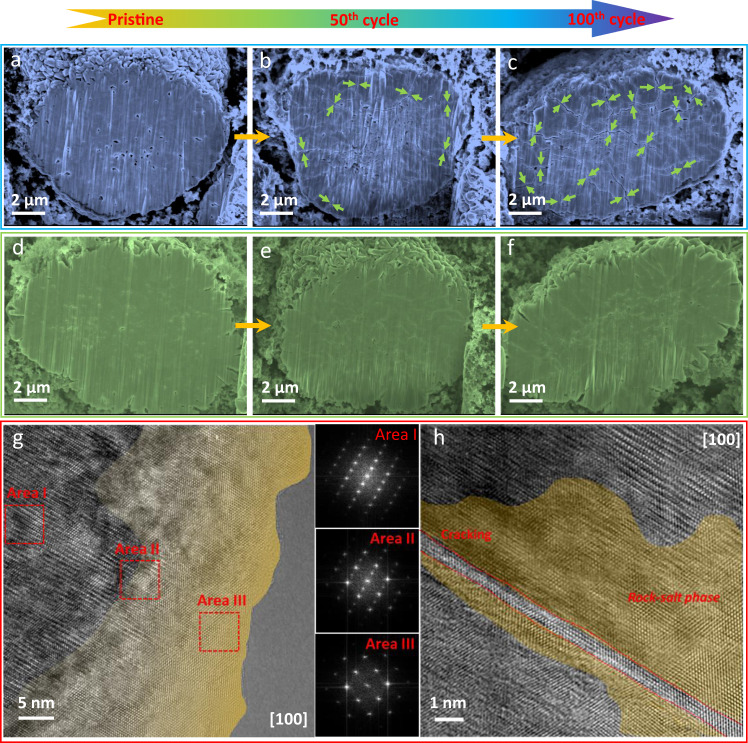
Ex situ postmortem FIB-SEM and TEM electrode characterizations of two gradient cathodes. **a**–**c** Cross-sectional images of pristine NC-NM82 particle, NC-NM82 after 50 cycles, and NC-NM82 after 100 cycles, respectively. The scale bar is 2 µm. **d**–**f** Cross-sectional images of pristine NM-NC82 particle, NM-NC82 after 50 cycles, and NM-NC82 after 100 cycles, respectively. The scale bar is 2 µm. **g**, **h** High-resolution TEM images of the cycled NC-NM82 surface area and the NC-NM82 cracking area. The scale bars are 5 and 1 nm, respectively.

To further correlate particle morphological stability with gradient composition design, ex situ postmortem focused ion beam-scanning electronic microscopy (FIB-SEM) and transmission electron microscopy (TEM) are carried out to visually observe morphological/structural evolution of the two gradient cathode materials. The cross-sectional image from FIB (Fig. 6a) shows that the pristine particle of NC-NM82 is densely packed with primary particles. Some microcracks started to appear on the NC-NM82 particle surface and gradually extend into the core after 50 cycles (Fig. 6b). Even worse, the microcracks continue to expand throughout the entire particle after 100 cycles (Fig. 6c). These cracks expose the particle core to electrolyte and exacerbate parasitic side reactions on not only the secondary-particle surfaces but also inside the primary particles. The global occurrence of parasitic side reactions in the NC-NM82 particles induce the rapid capacity loss and structure degradation, which is highly consistent with the in situ XRD and the d*Q*/d*V* results. The cross-sectional image of pristine NM-NC82 (Fig. 6d) exhibits the similar morphology with NC-NM82, but it presents more stable mechanical performances during the extended cycles (Fig. 6e). The particles remain intact and only very tiny veins are observed after 100 cycles (Fig. 6f). More specifically, the stable surface still serves as protection to prevent electrolyte infiltration and effectively suppresses the parasitic reaction occurring in the interior of the particles. This is also confirmed by the TEM observations. As shown in Supplementary Fig. 17, both intergranular and intragranular cracks were observed in the cycled NC-NM82 sample. High-resolution TEM image (Fig. 6g) shows that the surface structure has been transferred to the irreversible rock-salt phase due to parasitic side reactions between the highly oxidized particle surface and the electrolyte^31,34^. Since severe particle cracking manifests as illustrated in Fig. 6f and Supplementary Fig. 18, the fresh surface will be exposed to the electrolyte and trigger additional irreversible phase transition that extends into the bulk. Figure 6h confirms that irreversible phase transition similarly occurs in cracks inside the particles. Identical characterizations are conducted on NM-NC82 (Supplementary Fig. 19). TEM results demonstrate that irreversible phase transition is also present in the surface area of NM-NC82, which indicates that this destructive reaction is independent of Co and Mn compositions. However, particle cracking and extended rock-salt phase are absent from the bulk of NM-NC82. Observation of the boundary area (Supplementary Fig. 20) shows that the crystallinity of the layered structure remains stable. This further confirms that NM-NC82 gradient composition design benefits mechanical stability, mitigates particle cracking, and leads to improved electrochemical performances.

**Fig. 7:**
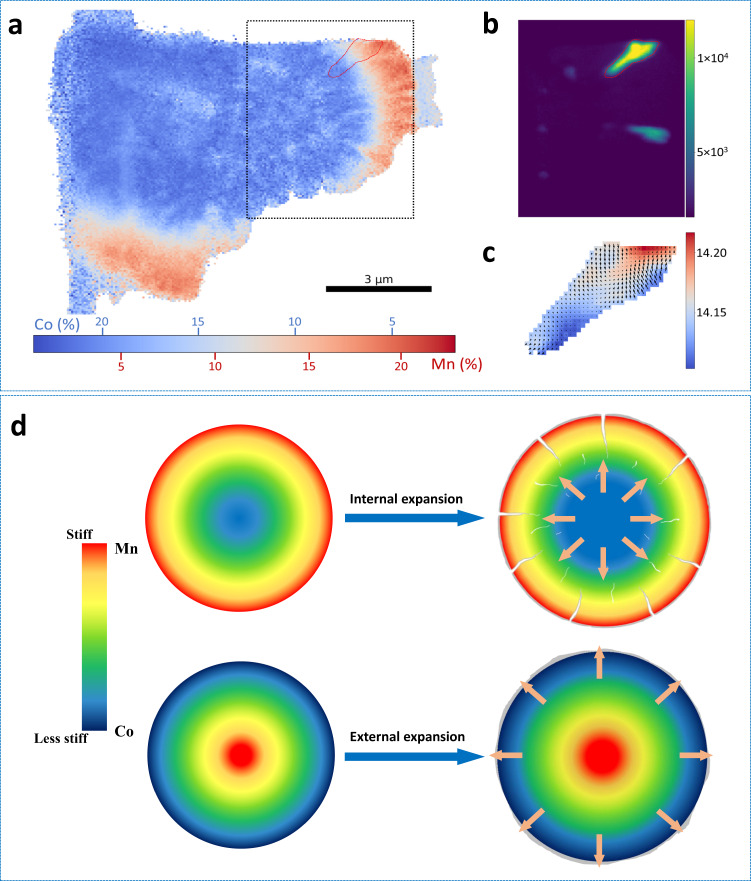
Nanoscale structure variation within concentration gradient cathodes and the relationship between concentration designs and mechanical properties. **a** Spatial element distributions of Mn (red) and Co (blue) in NC-NM82 sample, detected by X-ray fluorescence. The color bar refers to the concentration of Co and Mn. **b** X-ray nanodiffraction image of NC-NM82 sample. The color bar refers to the diffraction intensity. **c** Detailed analysis of nanodiffraction in the selected primary particle. The color bar refers to the lattice parameters. **d** The mechanism schematic of two different concentration gradient designs and their mechanical stability.

In order to understand the correlation between structural properties and concentration distribution, ex situ nanoscale XRF and scanning X-ray diffraction microscopy (SXDM) were performed to study the local composition and structure. The XRF result on the cross-section of a pristine NC-NM82 secondary particle prepared by FIB, with an obvious compositional gradient from a Co-rich core to a Mn-rich surface (Fig. 7a). Figure 7b shows the integrated, diffracted intensity at an arbitrary sample and a certain detector angle (corresponding to the [006] reflection). Two primary particles were visible under this diffraction condition. Figure 7c shows the *d*-spacing and lattice tilt map of the primary particle marked in Fig. 7b. The *d*-spacing map indicates that initial *c*-lattice parameter in the Mn-rich region is larger, which is consistent with the observation in NM82. Interestingly, inward lattice rotations were observed at the boundary of the outer-layer primary particles and were more pronounced closer to the surface. Lattice rotation is an effective mechanism to relax compressive stress when primary particles expand into each other during growth. This indicates that compressive stress mainly exists in the pristine particle surface. Areas on the surface undergo even more severe compressive stress when lattice expansion is generated with delithaition^19^. Likewise, the Co-enriched core composition undergoes greater volume changes/tension towards the surface. Together, these two detrimental processes significantly aggravate the mechanical degradation of the stiffer Mn-rich surface, which is evidenced by the generation of particle cracking observed in Fig. 6a–c. In turn, this result indicates that rational composition design with Co-enriched surface (lower stiffness) enhances morphology stability and suppresses particle cracking.

A schematic was used to summarize the underlying mechanism, shown in Fig. 7d. Previous simulations results revealed that the bulk structure of secondary particles suffers from severe tension stress, while the surface undergoes compressive stress^18,19^. This is particularly true in the case of our two concentration gradient cathodes. When designed with a Mn-rich surface and a Co-rich core, the greater internal volume change and tensile stress induced by (de)lithiation will be imposed on the stiffer surface components, which aggravates mechanical degradation in the form of particle cracking. Mechanical degradation together with the resulting parasitic side reactions will contribute to fast structure degradation and capacity fading. Rational gradient design with a Co-rich surface and a Mn-rich core shows significantly improved mechanical stability. The smaller internal expansion combines with the less stiff Co-enriched surface, which effectively suppress the generation of particle cracking and enhance the electrochemical performance of Ni-rich cathode materials. Based on these fundamental discoveries, we reveal the significant effects of Co and Mn on mechanical properties and build the correlation between composition design and morphology stability. These insights can serve as design principles to develop new concentration gradient NMC cathodes and single crystal NMC cathode, and will facilitate future discovery of structurally stable cathode materials in lithium-ion batteries.

## Discussion

In summary, we thoroughly isolated the intrinsic mechanical properties of Co and Mn content in Ni-rich cathodes. Based on their different roles on mechanical properties, we designed two comparable concentration gradient cathodes with variable Mn and Co content to investigate the correlation between composition design and mechanical stability. A combination of electrochemical tests, in situ HEXRD, ex situ postmortem cross-sectional TEM and SXDM revealed that the engineered gradient Mn/Co concentration has significant impacts on mechanical/structural stability and electrochemical performance. NM-NC82 benefits from lower stiffness in Co-enriched surfaces as it shows negligible morphology damage and improved stability of the *H*_2_–*H*_3_ phase transition during extended cycling. By contrast, NC-NM82 observes severe particle cracking caused by internal tensile stress and the stiffer surface. When combined with its degraded *H*_2_–*H*_3_ phase transition, the capacity loss is significantly accelerated. These apparent discrepancies between two converse concentration gradient designs re-emphasize the importance of mechanical properties and Co/Mn composition design in structural and morphology stability. This work provides new design principles for the concentration gradient cathodes and is beneficial for future discovery of structurally stable cathode materials in LIBs.

## Methods

### Materials synthesis

The secondary particle precursors of NC-NM82, NM-NC82, and NMC811 were all synthesized via the co-precipitation method. A 4 L batch reactor was employed to synthesize all precursors. Appropriate amounts of NiSO_4_·6H_2_O (Sigma-Aldrich, ≥98%), CoSO_4_·7H_2_O (Sigma-Aldrich, ≥99%), and MnSO_4_·H_2_O (Sigma-Aldrich, ≥99%) were used to prepare the 2.0 M starting solutions without any pre-treatments. For the precursor of NMC811, the starting solution mixture of Ni, Co, and Mn at 2.0 mol L^−1^ was pumped into the reactor under N_2_ atmosphere. For the NC-NM82 precursor, a starting solution mixture of Ni and Co stored in the first container was pumped into the reactor first; then, a solution mixture of Ni and Mn in the second container was pumped into the first container. A reverse order for adding solutions was used to prepare the NM-NC82 precursor. In addition to the transition metal solutions, 4.0 mol L^−1^ NaOH solution (aq.) and 5.0 mol L^−1^ NH_4_OH solution (aq.), which acted as precipitating agent and chelating agent, were also pumped into the reactor. During co-precipitation synthesis process, the pH (~11.0), temperature (∼60 °C), and stirring rate (1000 rpm) were carefully controlled. The precursor powders were obtained by filtering, washing, and vacuum drying in an oven overnight. NC-NM82, NM-NC82, and NMC811 were prepared by thoroughly mixing the corresponding precursors with appropriate contents of LiOH·H_2_O (Sigma-Aldrich, ≥99%, the molar ratio of Li:[Ni + Mn + Co] = 1.03:1). This was then followed by identical calcination conditions at 750 °C for 12 h under an oxygen atmosphere.

### Electrochemistry tests

For electrode preparation, active materials were mixed with carbon black (C45 Conductive Carbon Black, TIMCAL) and polyvinylidene fluoride (PVDF, 8%wt Solvay® 5130 PVDF binder dissolved in *n*-methyl-2-pyrrolidone (NMP)) at 80:10:10 wt% ratios and then followed by grinding the mixture in a mortar at 2000 r.p.m. for 9 min (3 min per time, a total of three times) in air atmosphere. The electrodes were dried at 80 °C under vacuum for 12 h to remove all traces of solvent. The 2032 type coin cells were used to prepare lithium metal cells. Celgard 2325 separators (25 µm), 1.2 M LiPF_6_ in EC/EMC (3:7) electrolyte (GEN II with a water content below 20 ppm, 40 µl), and Li metal foil (MTI, 16 mm × 0.6 mm (diameter × thickness), high purity of 99.9%) were used. The metal cells were then cycled between 2.8 and 4.4 V vs Li^+^/Li and carried out in the environmental chamber at 25 °C and the error of the temperature is ±2 °C.

### Synchrotron X-ray diffraction measurement

High-energy X-ray diffraction (HEXRD) was performed at the 11-ID-C beamline of the Advanced Photon Source at Argonne National Laboratory. A high-energy X-ray with a beam size of 0.2 mm × 0.2 mm and a wavelength of 0.1173 Å was used. Diffraction patterns were collected in the Laue diffraction geometry using a Perkin-Elmer area detector, placed at 1800 mm from the samples. Rietveld refinement of the collected HEXRD patterns were carried out using GSAS package.

In situ HEXRD measurements during cycling were performed on the same beamline. The high penetration and low absorption of HEXRD is beneficial to observe tiny phase changes that are usually invisible from lab scale XRD. The 2032-coin cells have a 3 mm hole suitable for X-rays to pass through and diffraction patterns were collected every 10 min. Kapton tape was used to seal the holes of the coin cells, preventing them from air exposure.

### Compositional and local structural characterization by synchrotron X-ray nanoprobe

3D synchrotron XRF mapping was performed at the Hard X-ray Nanoprobe Beamline of the National Synchrotron Light Source II at Brookhaven National Laboratory. The nanoprobe experiment was carried out at 12 keV using a 12-nm beam focused with a pair of multilayer Laue lenses^40^. The sample particles were drop-casted on a 10-µm-thick Si sample holder. There are patterned fiducial lines (Cr) on the Si holder for locating the sample, conducting focus, and rotation alignment. Tomography measurements were performed by collecting a total of 91 projections from −90° to 90°, with 2° intervals. The tomographic reconstruction was carried out using the algebraic reconstruction technique^41^.

2D XRF and SXDM were performed using the hard X-ray nanoprobe of the Center for Nanoscale Materials (CNM) at sector 26-ID-C of the Advanced Photon Source, Argonne National Laboratory. The X-ray energy was 10 keV. A Fresnel zone plate (150 μm diameter gold pattern, 16 nm outer zone, 400 nm thickness) focuses the X-ray beam to the size of 20 nm. The fluorescence signal was collected using a SII Vortex-ME4 SDD EDS 4-element detector. The scattering recorded on a pixel-array detector with 55 μm pixels located 900 mm from the sample (ASI Medipix). For the *d*-spacing and lattice tilt map, 2D raster scans were performed at 17 rocking angles around the 006 reflection of the primary particle, with an angular step size of 0.25°.

### Ex situ FIB-SEM and TEM measurement

The metal cells after electrochemical cycles were disassembled in an Ar-filled glove box. Subsequently, the cathode electrodes were washed immediately using dimethyl carbonate and then completely dried under vacuum. After that, the cathode electrodes were cut into small pieces and pasted on a sample stage of FIB-SEM. Lastly, the sample stage is sealed in a jar filled with Ar gas. When transferring the sample stage into the FIB-SEM instrument, the samples will be exposed to the air for a few seconds. The cross-sectional SEM observations were conducted by a Zeiss NVision 40 FIB-SEM dual-beam system. For TEM tests, thin-section TEM specimens were prepared from each electrode foils by standard FIB lift-out procedure. The specimens were first thinned to 200 nm by a 30 kV Ga ion beam, and then further polished with a 5 kV Ga ion beam to remove damage layer. TEM and HRTEM characterizations were conducted using the Argonne Chromatic Aberration-Corrected TEM (ACAT) (a FEI Titan 80–300 ST with an image aberration corrector to compensate for both spherical and chromatic aberrations) at an accelerating voltage of 200 kV.

## Supplementary information

Supplementary Information

## Data Availability

All relevant data that support the findings of this study are presented in the manuscript and supplementary information file. Source data are available from the corresponding author upon reasonable request.
