# Supplementary material for: Rational design of mechanically robust Ni-rich cathode materials via concentration gradient strategy
Source: Nat Commun. 2021 Oct 15;12:6024. doi: 10.1038/s41467-021-26290-z (PMC8520018; doi:10.1038/s41467-021-26290-z)
Supplement: Supplementary file 1 — Supplementary Information [file 41467_2021_26290_MOESM1_ESM.pdf]

Supplementary Information

Rational design of mechanically robust Ni-rich cathode materials via concentration gradient strategy

Tongchao Liu[1,6], Lei Yu[2,6], Jun Lu[1,], Tao Zhou[2], Xiaojing Huang[3], Zhonghou Cai[4], Alvin Dai[1], Jihyeon Gim[1], Yang Ren[4], Xianghui Xiao[3], Martin V. Holt[2], Yong S. Chu[3], Ilke Arslan[2], Jianguo Wen[2,*], Khalil Amine[1,5,*]*

[1]*Chemical Sciences and Engineering Division, Argonne National Laboratory, Lemont, IL, 60439, United States*

[2]*Center for Nanoscale Materials, Argonne National Laboratory, Lemont, IL 60439, USA*

[3]*National Synchrotron Light source II, Brookhaven National Laboratory, Upton, NY 11973, USA*

[4]*X-ray Science Division, Advanced Photon Sources, Argonne National Laboratory, Lemont, Illinois, 60439, United States*

[5]*Material Science and Engineering, Stanford University, Stanford, CA 94305, USA*

[6]*These authors contributed equally to this work.*

*Corresponding author: amine@anl.gov (K. A.); junlu@anl.gov (J. L.); jwen@anl.gov (J. W.);

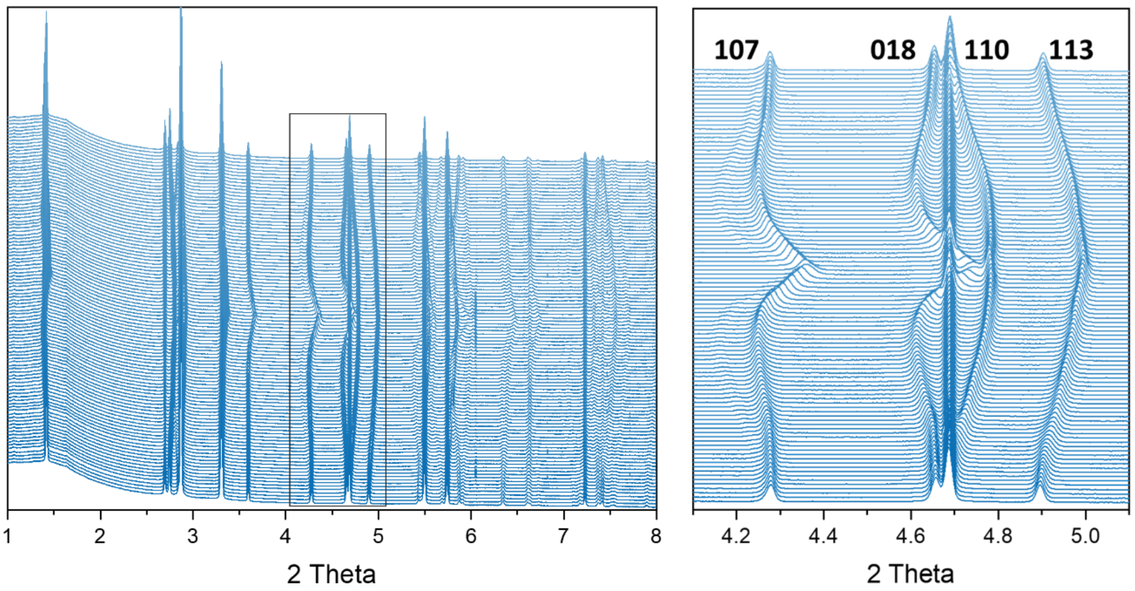

Supplementary Figure 1. The in situ XRD patterns of NC82 during the first charge/discharge in the voltage range of 2.8-4.5 V using a current rate of C/10 (1C = 200 mA g^{-1}). The obvious phase transitions and lattice parameter changes can be observed from in situ XRD patterns, particularly in the 2-theta range of 4.0-5.0.

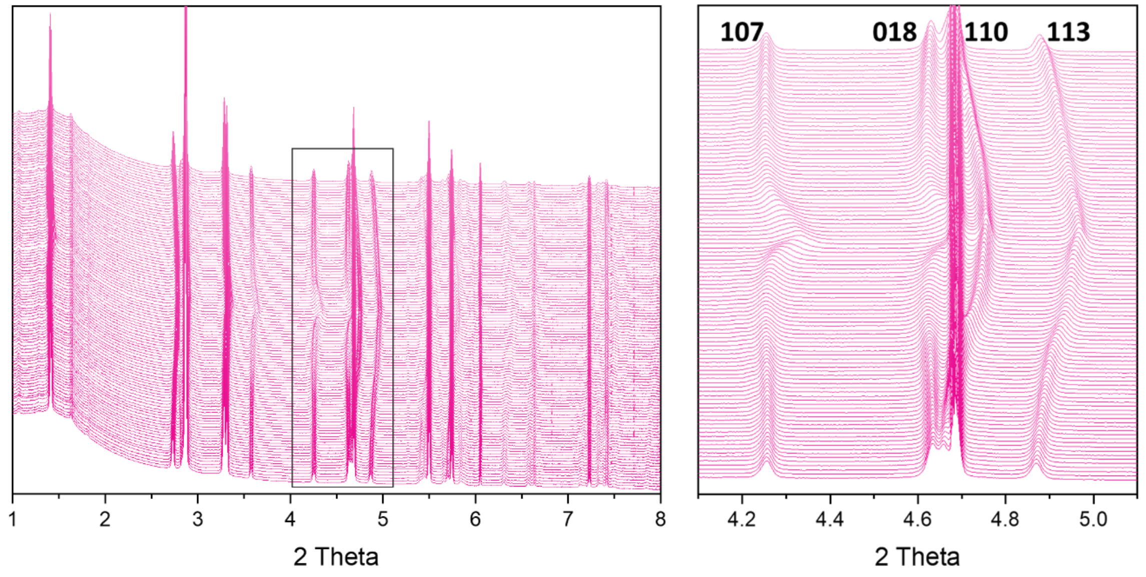

Supplementary Figure 2. The in situ XRD patterns of NM82 during the first charge/discharge in the voltage range of 2.8-4.5 V using a current rate of C/10 (1C = 200 mA g^{-1}). The obvious phase transitions and lattice parameter changes can be observed from in situ XRD patterns, particularly in the 2-theta range of 4.0-5.0. Compared to NC82, NM82 shows smaller lattice parameter changes when operated at the same conditions.

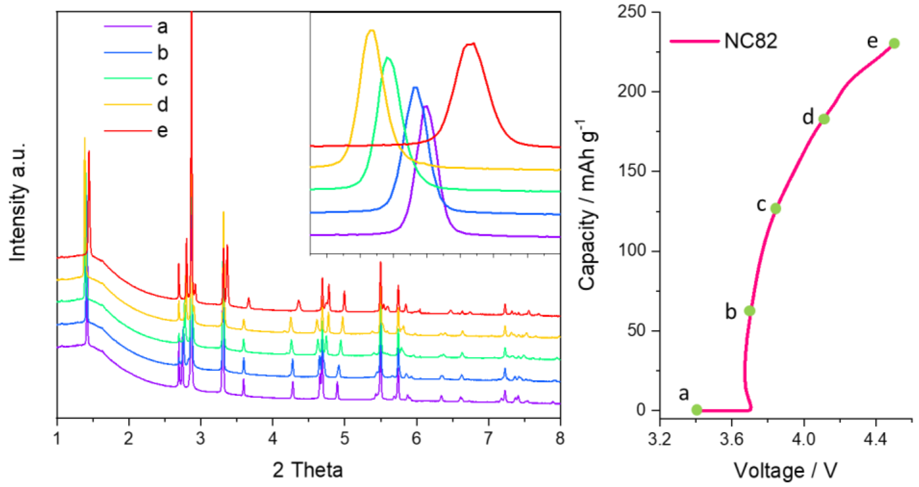

Supplementary Figure 3. Ex situ HEXRD measurement and the corresponding electrochemical curve of NC82 samples. The cells were operated at a current rate of C/10 and stopped at the SOCs of 0, 20%, 40%, 60% and 82%, respectively. The insert image is the enlarged XRD patterns for [003] reflection. The obvious lattice parameter changes along the c axis can be observed from ex situ XRD patterns.

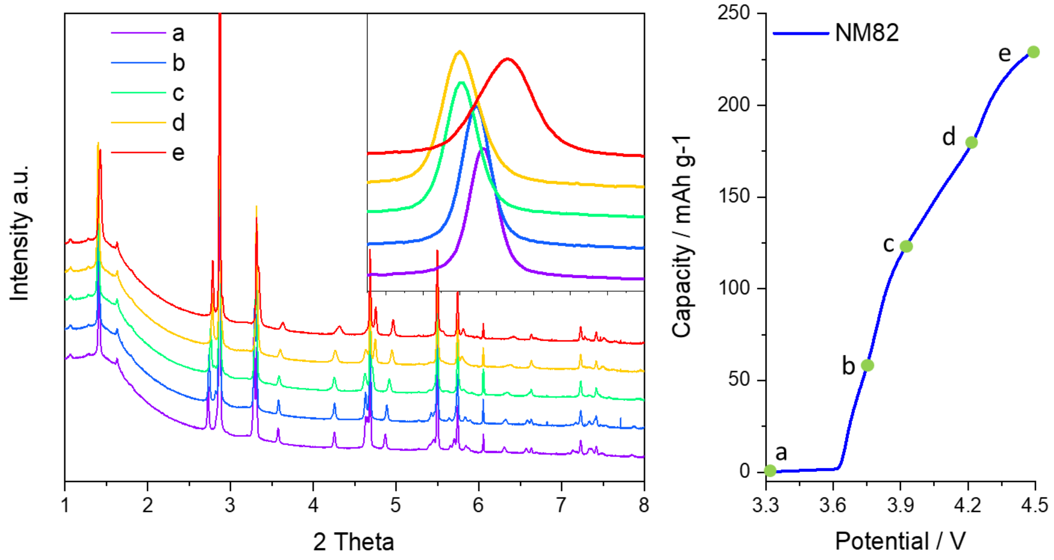

Supplementary Figure 4. Ex situ XRD measurement and the corresponding electrochemical curve of NM82 samples. The cells were operated at a current rate of C/10 and stopped at the SOCs of 0, 20%, 40%, 60% and 80%, respectively. The insert image is the enlarged XRD patterns for [003] reflection. The obvious lattice parameter changes along the c axis and peak broadening can be observed from ex situ XRD patterns.

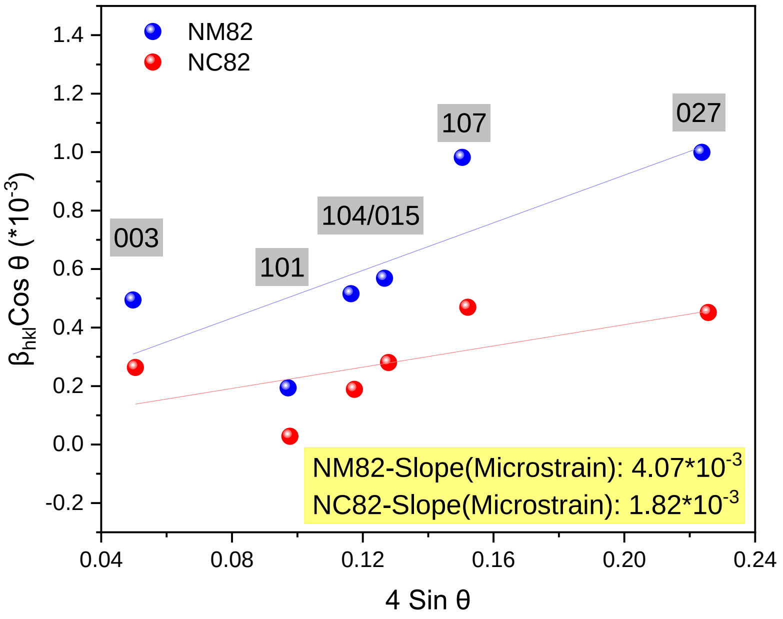

Supplementary Figure 5. Williamson-Hall plots of NM82 and NC82 samples charged to 4.5V with diffraction peaks from (hkl) planes. The linear fitting errors of these two samples are $9.3*10^{-4}$ and $6.4*10^{-4}$.

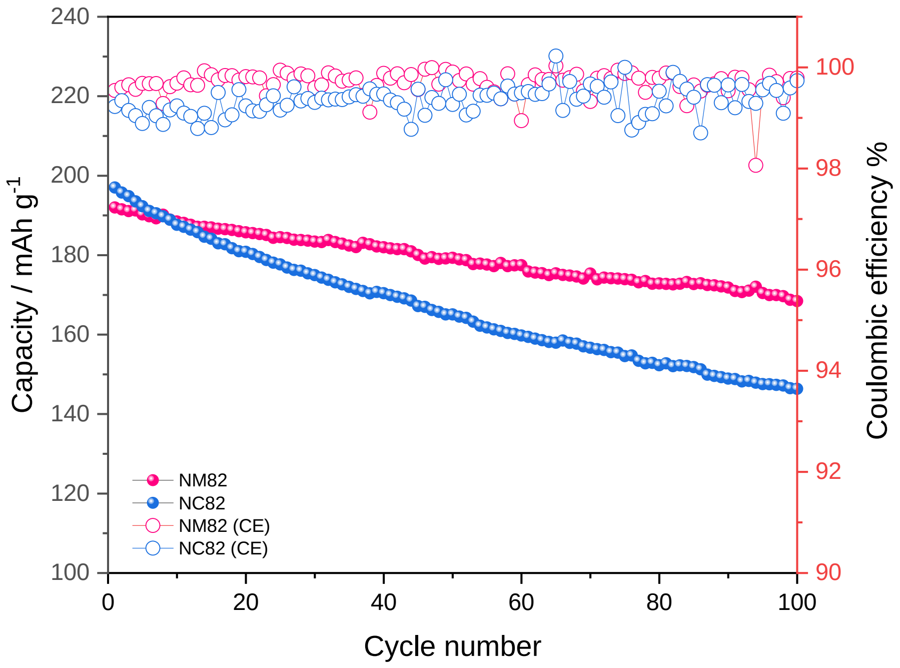

Supplementary Figure 6. Cycle performance comparison of NC82 and NM82 at a current rate of C/3 within a voltage range of 2.8 - 4.5 V.

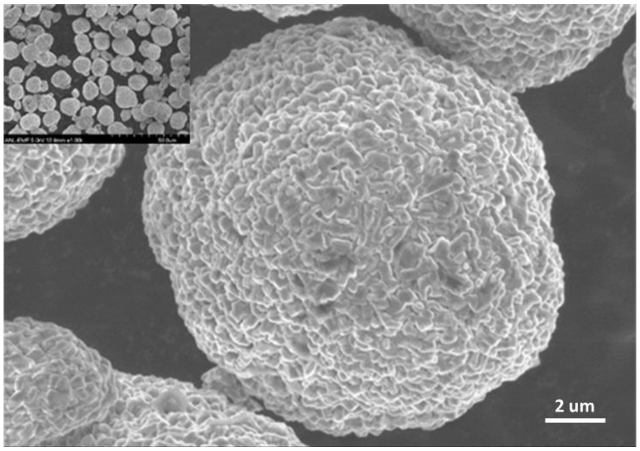

Supplementary Figure 7. Morphology characterization for NMC811 by SEM. The average particle size of NMC811 is around 12 μm.

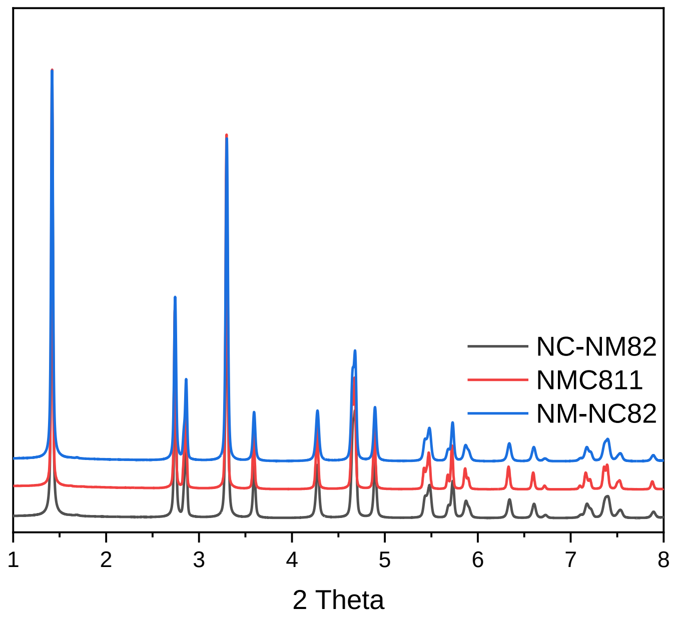

Supplementary Figure 8. High energy XRD curves of pristine NC-NM82, NMC811, and NM-NC82.

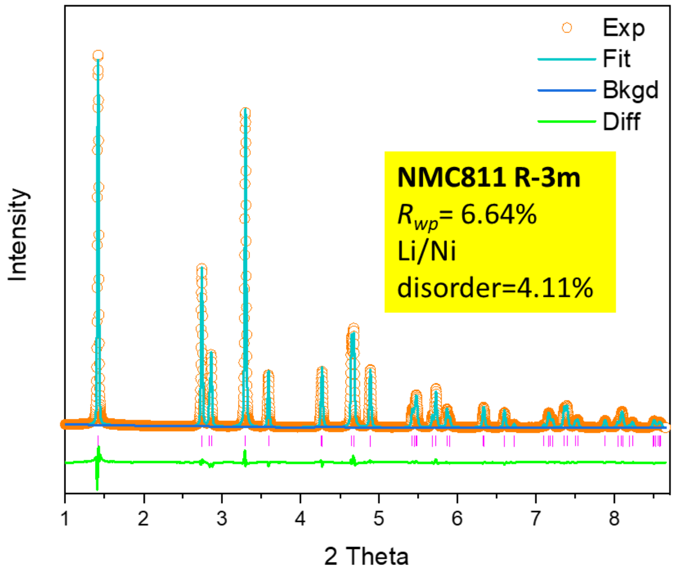

Supplementary Figure 9. High-energy XRD curves and Rietveld refinement result of NMC811. The Li/Ni disorder in NMC811 is 4.11%.

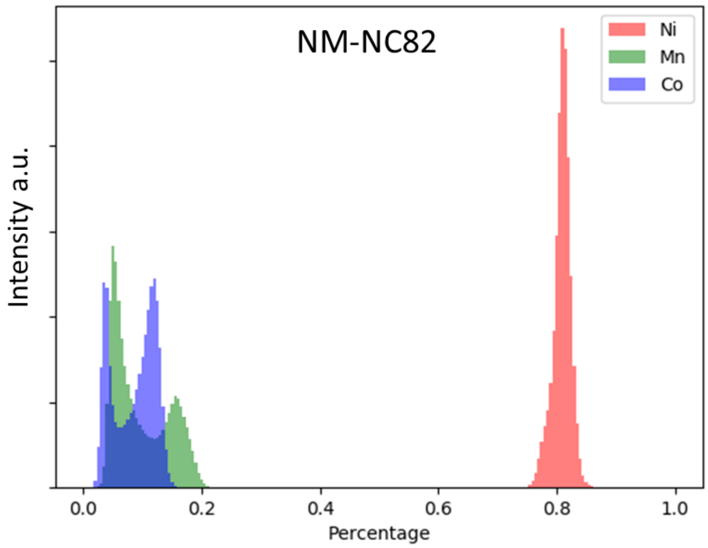

Supplementary Figure 10. Quantitative composition analysis for NM-NC82 by 3D X-ray fluorescence.

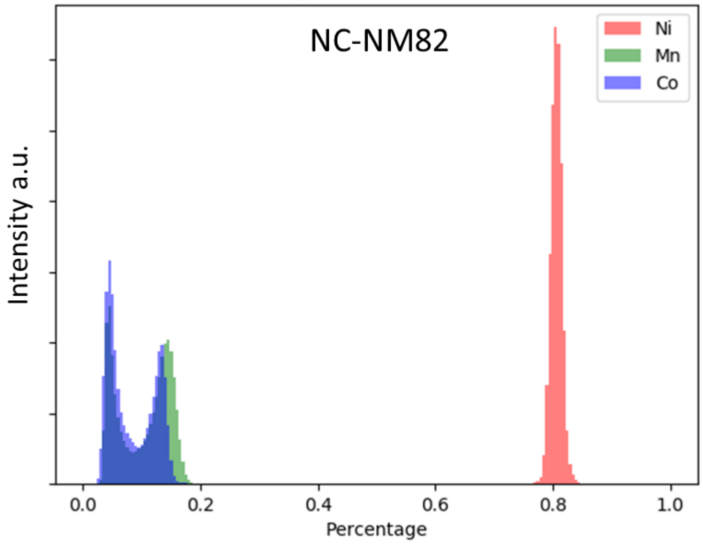

Supplementary Figure 11. Quantitative composition analysis for NC-NM82 by 3D X-ray fluorescence.

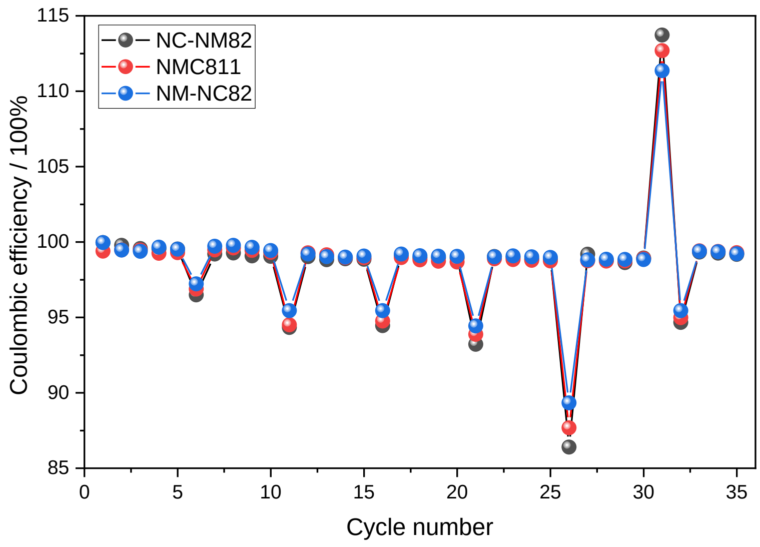

Supplementary Figure 12. The corresponding coulombic efficiency of the rate performance test in **Figure 4b.**

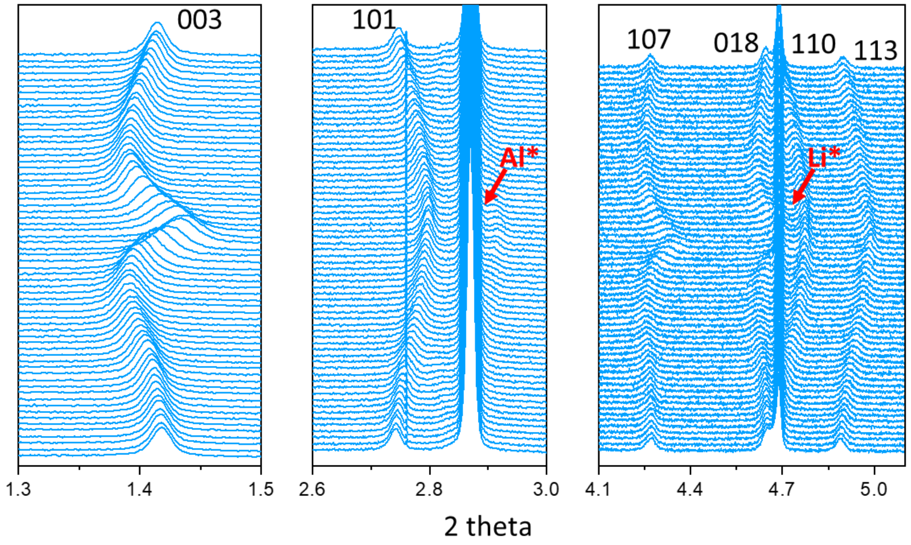

Supplementary Figure 13. In situ XRD patterns of NC-NM82 during the first charge/discharge in the voltage range of 2.8-4.4 V using a current rate of C/10 (1C = 200 mA g^{-1}). The obvious phase transitions and lattice parameter changes can be observed from in situ XRD pattern.

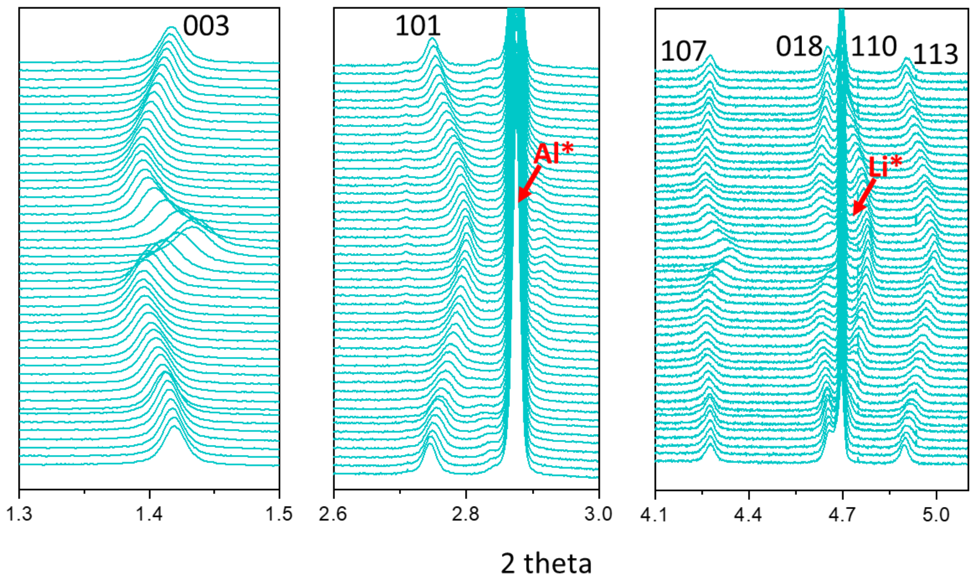

Supplementary Figure 14. In situ XRD patterns of NM-NC82 during the first charge/discharge in the voltage range of 2.8-4.4 V using a current rate of C/10 (1C = 200 mA g^{-1}). The obvious phase transitions and lattice parameter changes can be observed from in situ XRD pattern.

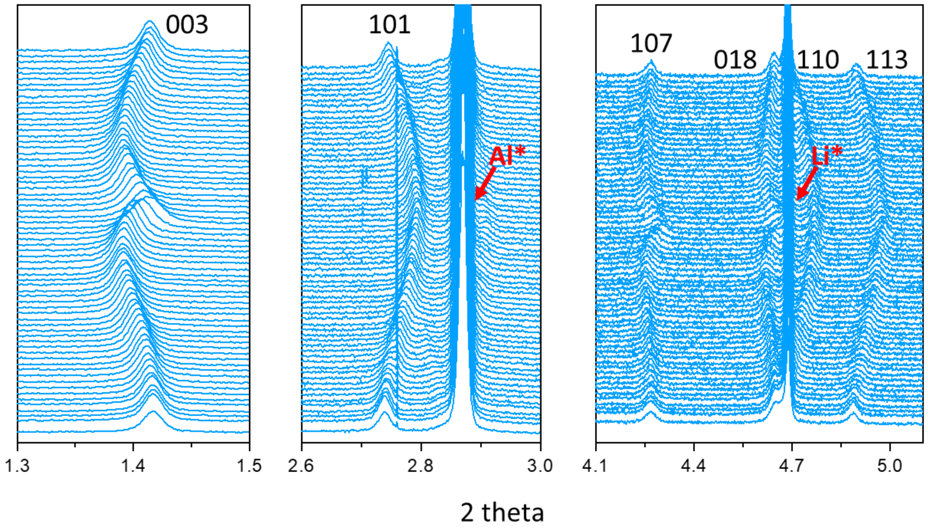

Supplementary Figure 15. In situ XRD patterns of NC-NM82 during the 101th charge/discharge in the voltage range of 2.8-4.4 V using a current rate of C/10 (1C = 200 mA g^{-1}). The phase transitions and lattice parameter change significantly degrades after 100 cycles.

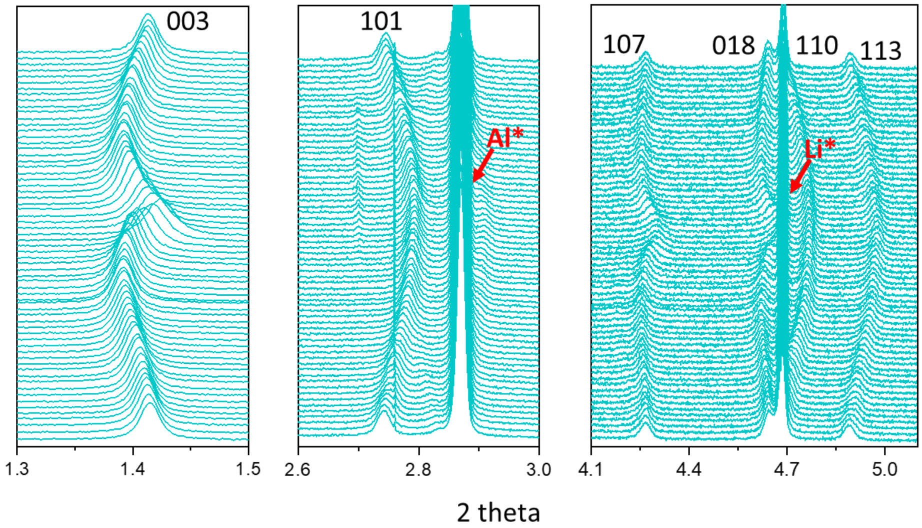

Supplementary Figure 16. In situ XRD patterns of NM-NC82 during the 101th charge/discharge in the voltage range of 2.8-4.4 V using a current rate of C/10 (1C = 200 mA g^{-1}). The phase transitions and lattice parameter changes slightly decay after 100 cycles.

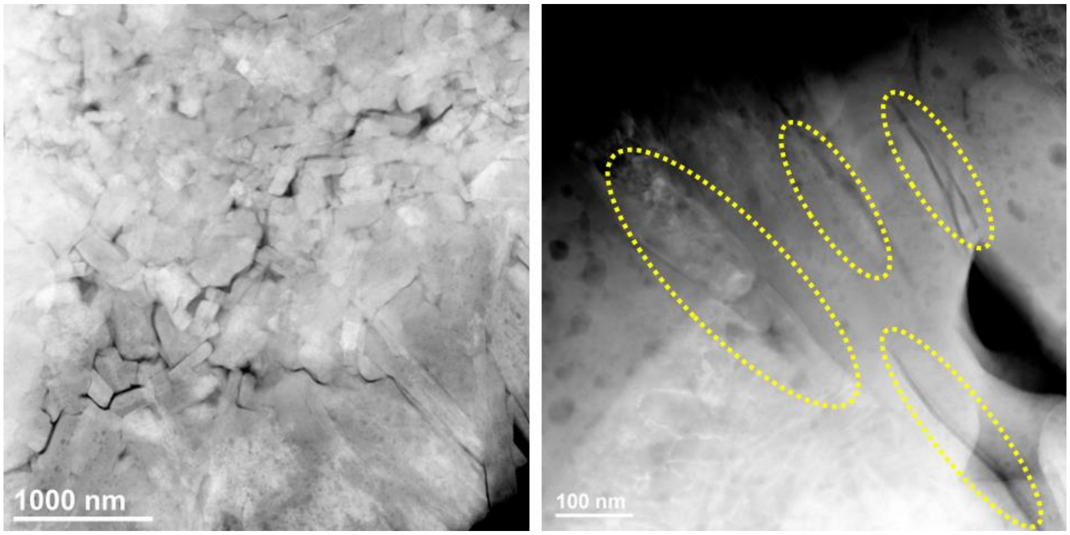

Supplementary Figure 17. Ex situ postmortem low magnification STEM-HAADF images of NC-NM82 particle after 100 cycle at a current rate of C/2. Particle cracks occur in the particle interior and particle surface area.

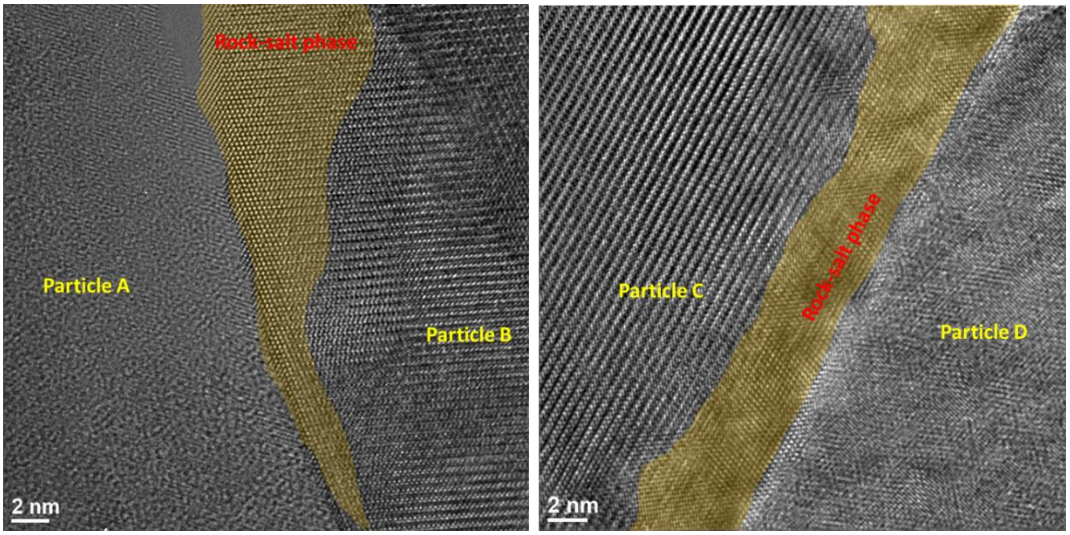

Supplementary Figure 18. Ex situ postmortem high magnification TEM images of NC-NM82 sample after 100 cycles at a current rate of C/2. It is evident that the structures at cracking areas change to the rock-salt phase.

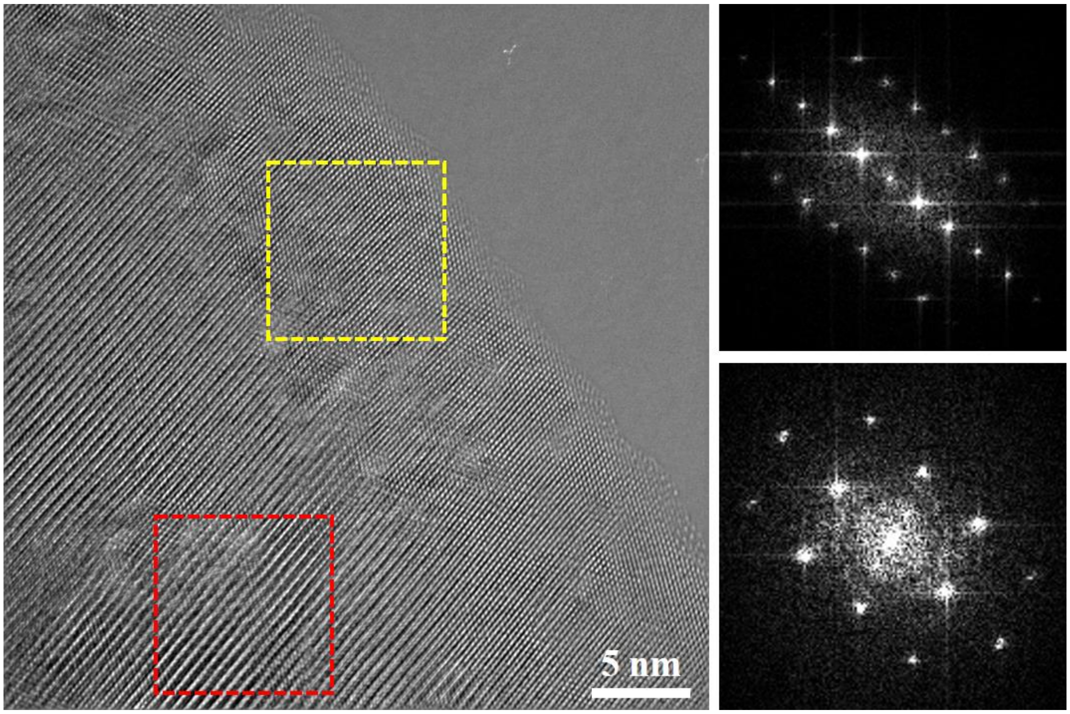

Supplementary Figure 19. Ex situ postmortem high resolution TEM observation of the surface structure of NM-NC82 sample after 100 cycles at a current rate of C/2. The image on the right-top corner is the corresponding fast fourier transform (FFT) pattern of the region marked by yellow box. The image on the right-bottom corner is the corresponding FFT pattern of the region marked by red box. This result shows the structures of the surface area change to the rock-salt phase.

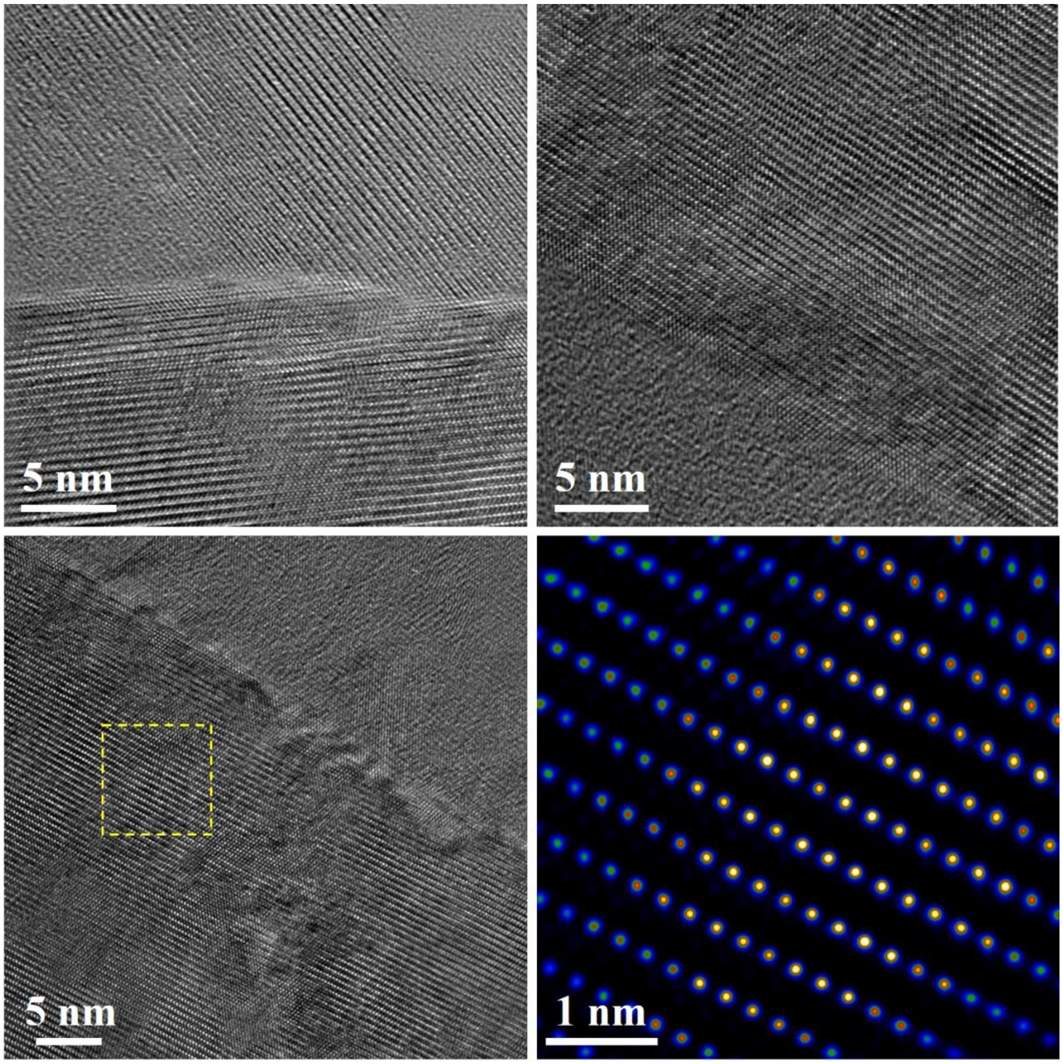

Supplementary Figure 20. Ex situ postmortem high resolution TEM observation of the boundary area of NM-NC82 after 100 cycles at a current rate of C/2. The image on the right-bottom corner is the enlarged area marked by yellow box. The structure at the grain boundary area remains stable, indicating the electrolyte cannot penetrated into the particle interior without particle cracks.

Supplementary Table 1. Inductively coupled plasma-atomic emission spectrometry results of NM-NC82, NMC811, and NC-NM82. All element contents are normalized with the nickel content.

Samples	Li	Ni	Mn	Co
NM-NC82	1.006	0.800	0.101	0.098
NMC811	1.004	0.800	0.100	0.101
NC-NM82	1.003	0.800	0.099	0.102

Supplementary Table 2. Refinement results of the HEXRD measurements of NC-NM82, NMC811 and NM-NC82, including lattice parameters, atomic occupancy and R_{wp}.

Samples	*a=b*	*c*	Li/Ni disorder	R_{wp}
NC-NM82	2.8711(2) Å	14.1931(4) Å	3.1(5)%	4.27%
NMC811	2.8712(4) Å	14.1987(3) Å	4.1(1)%	6.64%
NM-NC82	2.8718(1) Å	14.2001(3) Å	3.3(5)%	4.68%

Sample	Atom	Mult	Fractional coordinates			Occupancy	Uiso
NC-NM82	Li1	3	0	0	0	0.968(5)	0.0071(7)
	Ni2	3	0	0	0	0.031(5)	0.0071(7)
	Ni1	3	0	0	0.5	0.768(5)	0.0059(9)
	Mn1	3	0	0	0.5	0.1000	0.0059(9)
	Co1	3	0	0	0.5	0.1000	0.0059(9)
	Li2	3	0	0	0.5	0.031(5)	0.0059(9)
	O1	6	0	0	0.2590(7)	1	0.0085(8)
NMC811	Li1	3	0	0	0	0.958(9)	0.0068(1)
	Ni2	3	0	0	0	0.041(1)	0.0068(1)
	Ni1	3	0	0	0.5	0.758(9)	0.0049(6)
	Mn1	3	0	0	0.5	0.1000	0.0049(6)
	Co1	3	0	0	0.5	0.1000	0.0049(6)
	Li2	3	0	0	0.5	0.041(1)	0.0049(6)
	O1	6	0	0	0.2591(5)	1	0.0073(2)
NM-NC82	Li1	3	0	0	0	0.966(5)	0.0047(9)
	Ni2	3	0	0	0	0.033(5)	0.0047(9)
	Ni1	3	0	0	0.5	0.766(5)	0.0055(2)
	Mn1	3	0	0	0.5	0.1000	0.0055(2)
	Co1	3	0	0	0.5	0.1000	0.0055(2)
	Li2	3	0	0	0.5	0.033(5)	0.0055(2)
	O1	6	0	0	0.2591(3)	1	0.0084(9)
